# The Impact of COVID-19 on Academic Performance and Personal Experience Among First-Year Medical Students

**DOI:** 10.1007/s40670-022-01537-6

**Published:** 2022-03-21

**Authors:** Shaun Andersen, Genesis Leon, Deepal Patel, Cynthia Lee, Edward Simanton

**Affiliations:** Kirk Kerkorian School of Medicine at UNLV, Las Vegas, NV USA

**Keywords:** COVID-19, Medical education, Online learning, Medical students, Academic performance

## Abstract

**Background:**

The COVID-19 pandemic forced medical education to rapidly transition from in-person learning to online learning. This change came with learning difficulties, social isolation, limited student/faculty relationships, and decreased academic performance.

**Objective:**

The purpose of this study is to determine if academic performance, study habits, student/faculty relationships, and mental health were different in first-year medical students (class of 2024) during the COVID-19 pandemic compared to pre-COVID cohorts.

**Methods:**

In April 2021, a survey was sent to first-year medical students at the Kirk Kerkorian School of Medicine at UNLV asking them to reflect on their experiences during the COVID-19 pandemic including study environment, mental health, and relationships with peers and faculty. A similar survey was sent to second- and third-year medical students (classes of 2023 and 2022) asking them to reflect on similar experiences during their first year of medical school. Exam scores for the first five exams were gathered and compared between first-, second-, and third-year medical students.

**Results:**

One hundred twenty-five students responded to the survey (81% of first-year students, 75% of second-year students, and 55% of third-year students). During the COVID-19 pandemic, first semester students did not score above the national average as much as first semester students pre-COVID (55% vs. 77%). Students during the pandemic studied at home more than previous cohorts. Mental health and relationships were all rated significantly lower among first semester students during the COVID-19 pandemic.

**Conclusions:**

Significant differences were found in first semester student experience and academic performance during the pandemic compared to pre-COVID cohorts.

## Introduction

The COVID-19 pandemic forced medical education to quickly pivot from traditional, in-person learning to an online format [1]. Most incoming first-year medical students usually have a difficult transition to make as they begin medical school. With the pandemic, they now had to face the unique challenge of abrupt changes to the structure of their education, as schools were transitioning and testing different methods of learning [2, 3]. While many impacts of these changes are still unknown, medical students have shown a range of attitudes toward remote and online learning. Some students have negative feelings toward online learning due to the perceived decline in education quality, lack of engagement, home distractions, and technical issues [4–7]. Other students appreciated advantages of online learning including increased flexibility, less commuting time, and freedom to learn at their own pace [4–7].

Exam scores are an important measure of mastering material in medical school, even during a pandemic. A study by Syed et al. evaluated the effects of the COVID-19 pandemic on academic performance in 79 first-year medical students by comparing pre-COVID grades with grades during COVID [8]. They found that students are still able to perform well academically and that no significant difference existed in students’ final grades. This work evaluates exam performance within a cohort affected by the COVID-19 pandemic. However, it does not compare exam performance between different cohorts at the same point in their medical education with or without the impact of COVID-19. Another study suggests that no significant difference occurs in exam performance between non-COVID and COVID cohorts [9]. While this study compares exam scores between cohorts, it only assesses two exam scores from a single organ system course. Since many medical schools were continuously making changes to their curriculum during the pandemic, assessing exam scores across multiple courses would be a better representation of COVID’s impact.

Studies show that medical students felt detached from their friends and families during the COVID-19 pandemic [10, 11]. Medical students’ mental health deteriorated significantly when comparing COVID with pre-COVID cohorts [12]. Slivkoff et al. surveyed first-year medical students regarding their mental health concerns and methods they were using to maintain wellness [2]. Students started meditating, pursued other hobbies, and exercised to maintain positive mental health. Although these studies assess COVID-19’s impact on mental health among medical students, they do not investigate if academic performance was impacted.

This study focuses on comparisons in study habits, mental health, student/faculty relationships, and exam scores between the cohort that started medical school during the pandemic and the cohorts pre-pandemic.

## Methods

### Subjects + Setting

The Kirk Kerkorian School of Medicine at UNLV admitted its first class in 2017. Traditionally, first-year medical students begin with the Emergency Response and Population Health (ERPH) course. In the ERPH, students practice basic life support and other patient care skills. The course also includes a population health component in which student groups are assigned to specific Las Vegas neighborhoods where they work on population health projects. These community service projects are designed for students to better understand their patient’s communities, what factors impact their health, and what resources are available to them. During this time, students get to know their cohort, socialize, build teams, instill trust, and ultimately forge strong relationships to obtain transferable skills that will assist them when facing new challenges in their medical training. As first-year students move into their didactic studies, they begin problem-based learning (PBL) sessions as well as live lectures. PBL is a method of learning where students aim to solve practical problems, diagnose, and treat medical cases on paper. Live lectures are taught by faculty and cover material from the preclinical science unit the students are in at that time. Each unit lasts 3–4 weeks and is concluded by a closed-book summative exam evaluated on a traditional letter grade system. The exam questions are chosen by administrators from a large question bank supplied by the National Board of Medical Examiners (NBME) with tested concepts staying consistent year-to-year. Live lectures and PBL meetings on campus encouraged students to congregate in the library and other study locations.

### Course Adjustments

During the COVID-19 pandemic, the ERPH was postponed, and the population health projects were completed virtually over an 18-month span. Although the ERPH course is typically the first course that students take, it is not foundational for the core science courses that follow. Therefore, the administration felt it could be appended to the didactic curriculum. First-year medical students (Class of 2024) immediately began the Introduction to Medical Sciences (IMS) course which previous cohorts had not begun until the conclusion of the ERPH. The IMS course also differed significantly from previous cohorts. Differences included remote PBL and a hybrid of online learning made up of synchronous and asynchronous lectures. The rapid shift to online formats also challenged faculty to present information online and virtually, often with many technical difficulties.

Because all instruction was virtual, most students did not travel to campus and consequently failed to bond with each other and faculty as had previous cohorts. As time passed, faculty noticed differences in student–faculty and student–student interactions compared to previous cohorts. Although the schedule and style of summative exams were not adjusted for the COVID cohort, faculty noticed differences in average scores on exams compared to previous cohorts. This concern led school leaders to collect data to try to understand the factors that might be leading to these differences in performance and interpersonal relationships.

### Data Sources

De-identified data were obtained from program evaluation databases in accordance with an approved Institutional Review Board (IRB) protocol. The two primary sources were an exam performance database and a survey sent to first-, second-, and third-year students intended to help school leaders better assess the impacts of the institutional reaction to the COVID-19 pandemic. First-year medical students received an electronic survey via Qualtrics that elicited information regarding their first semester experiences (during the COVID-19 pandemic). Second and third-year medical students (class of 2023 and 2022) received a similar survey; however, this version asked them to respond from the perspective of their first semester of medical school (before the pandemic). The surveys asked students about their experiences with study environments, mental health, student/faculty relationships, and other aspects of their first semester of medical school. These surveys also included open-response items asking students to elaborate on any of the previous survey items and to discuss how they felt COVID-19 impacted their experience. Open-response items were reviewed and organized into themes. Quotes from these themes are shown in the Results section. Response rates were 47/58 for the first-year students, 45/60 for the second-year students, and 33/60 for the third-year students.

### Statistical Analysis

NBME exams used in the study were constructed using the customized examination service (CAS). Items available for use in the CAS system previously used step 1 questions, and the mean performance for each item is provided by the CAS system. For each exam, the NBME provides a mean score for the items on that exam from when those same items were used on step 1 which schools can use as a comparison for the performance of their own students who took the exam. In this study, students who scored higher than the mean step 1 score provided by the NBME were coded as one (1). Students who scored below the provided NBME mean were coded as zero (0). Each of the cohorts included in the study took 5 similar exams (same general content) in their first semester of medical school, and the score used is a mean of the 5 exams as coded (1 = above step 1 mean, 0 = below the mean). Since the exams were slightly different with some being slightly easier or harder, this method of coding in relation to the step 1 mean was used to control for these small variations in test difficulty. The performance variable (NBME-alpha) was a mean of those five ratings. Group comparisons between COVID and non-COVID student groups were calculated using independent sampled *t* tests.

## Results

Mean comparisons of COVID and non-COVID groups are shown in Table 1. Descriptive statistics (mean, SD, *p* value) from the pre-COVID and COVID groups were calculated from survey responses to determine if either varied significantly in their experiences and to what degree. Both groups, first semester during COVID and non-COVID, were analyzed among each measure by way of Levene’s test for equality of variances. Statistically significant differences emerged from the following measures: NBME-alpha (*p* < 0.01), time spent studying at home (*p* < 0.01), time spent studying at campus (*p* < 0.01), time spent solo studying (*p* < 0.01), student relationship to faculty (*p* < 0.01), and student relationship to students (*p* < 0.01). Only ratings of mental health (*p* < 0.09) were not rated significantly different among both groups.

**Table 1:** Stratifying the surveyed population based on direct COVID impact

Measure	Class	*N*	Mean	Std. Deviation	*P* value
NBME-α (%)	Non-COVID	78	77.31	0.245	< 0.001
	COVID	47	54.89	0.269	
Time spent studying at home (%)	Non-COVID	78	57.12	31.824	< 0.001
	COVID	47	87.00	19.984	
Time spent studying at campus (%)	Non-COVID	78	28.73	27.685	< 0.001
	COVID	47	9.81	16.391	
Time spent solo studying (%)	Non-COVID	78	80.79	20.375	0.006
	COVID	47	88.91	12.397	
Mental health (1–5)	Non-COVID	78	3.44	1.112	0.089
	COVID	47	3.06	1.275	
Relationship to faculty (1–5)	Non-COVID	78	3.29	1.175	< 0.001
	COVID	47	1.79	0.778	
Relationship to students (1–5)	Non-COVID	78	4.05	1.005	< 0.001
	COVID	47	2.36	0.792	

Results are tabulated and shown in Table 1.

**Fig. 1:**
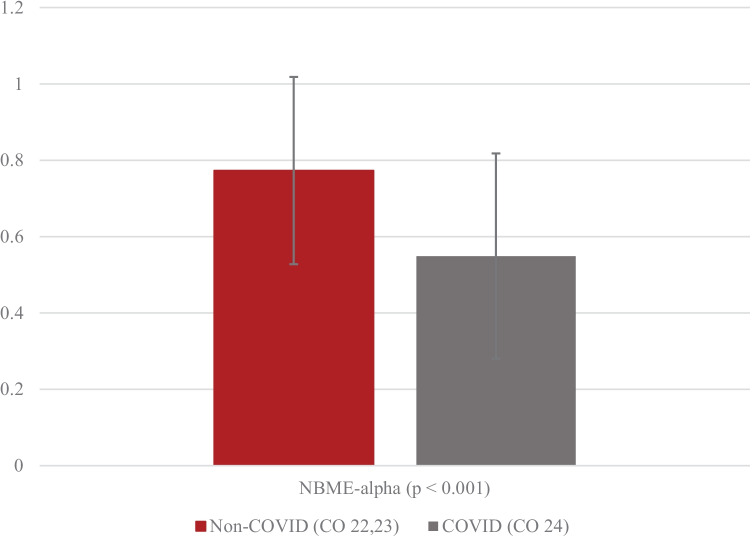
Group average performance on NBME examinations

To examine the effects of remote learning during the COVID-19 pandemic, we compared both groups against each other on the academic performance metric. Mean scores of NBME exams of non-COVID and COVID cohorts are shown in Fig. 1 on the *y*-axis.

First semester medical students during COVID scored above the national average on 54.9% of NBME exams versus first semester students during non-COVID years (77.3%). This 22.4% reduction in exam performance for the COVID cohort is reflective of significantly poorer performance across all five administered exams during the first semester time frame:

CO 24 Student: “I do not feel I’m learning as effectively, and I think that not being on campus and physically present for the curriculum has led to some habits that in the long run aren’t very sustainable in terms of solidifying and mastering the material.”

**Fig. 2:**
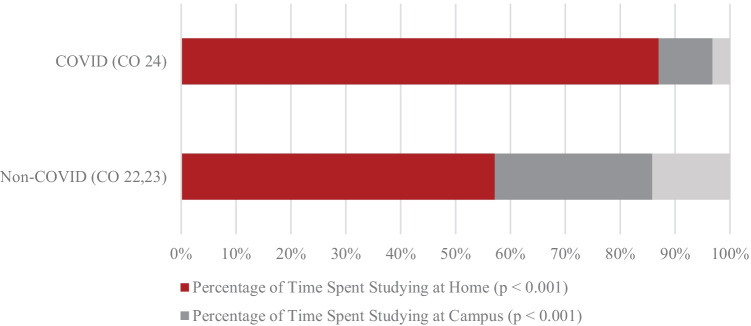
Group average time spent studying by location breakdown

To examine if the COVID-19 pandemic had significant impact on incoming medical student study location preference versus the non-COVID group, survey questions included prompts to allot total time spent studying between home and campus. Any response not totaling 100% among those two categories was considered to have an “elsewhere” location not otherwise specified (Fig. 2).

Figure 2 indicates that first-year students during COVID spent far less time studying on campus than second- and third-year students did (9.8% vs 28.75%):

CO 24 Student: “My first year of medical school has been an all-around awful experience, 100% due to the virtual reality of COVID. I barely know most of my classmates.”

They also spent more time at home studying (87.0% vs 57.1%). There is a clear at-home predominance for the COVID group, with little room for other locations not otherwise specified. The non-COVID group demonstrated a much more balanced distribution of study locations altogether:

CO 24 Student: “Studying alone in your room day in and day out wears down on you over time.”

**Fig. 3:**
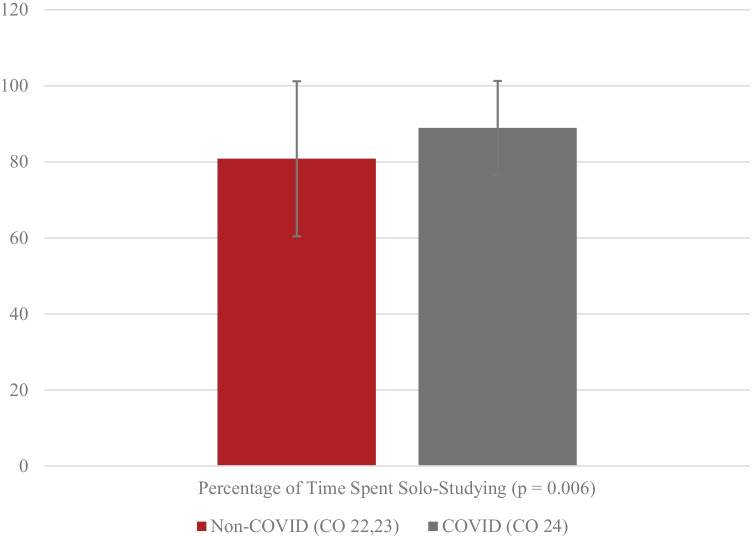
Group average time spent studying alone

We also aimed to assess if the COVID group shifted their study habits to more isolated studying relative to the non-COVID group. This would be doubly expected due to the effects of isolation during the pandemic and virtual education as a whole. Survey responses asking students to approximate their time spent studying alone by percentage were averaged among groups and are shown in Fig. 3.

According to Fig. 3, the COVID group spent more time studying alone than the non-COVID group did (88.9% vs 80.8%). This is in line with expectations set by Fig. 2, demonstrating preferences of the COVID group for not only studying at home but, by extension, alone as well.

**Fig. 4:**
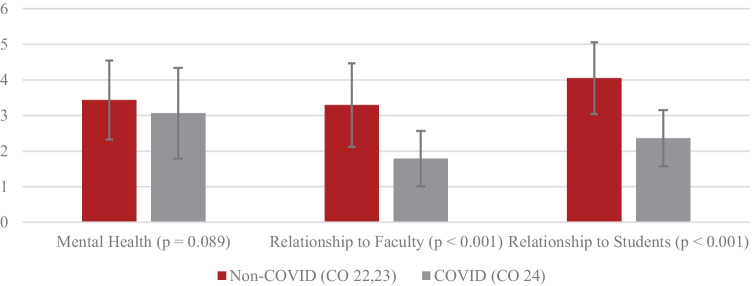
Group average mental health and relationship ratings

Finally, we assessed ratings of mental health, relationships with faculty and relationships with students on a 1–5 Likert scale to provide qualitative insight into student experience. Resulting averages and comparisons are shown in Fig. 4 with rating shown on *y*-axis.

Average mental health ratings were not statistically different between both cohorts (3.1/5 vs 3.4/5). Relationship ratings were all significantly lower among first semester students during COVID compared with the first semester students prior to COVID (relationship to faculty 1.8/5 vs 3.3/5) (relationships to students 2.4/5 vs 4.1/5):

CO 24 Student: “Very negatively. I’m someone who learns best from discussing in a small group and I live alone, so being at home was very isolating.”

CO 24 Student: “It has made other things I like doing outside of school either limited or non-existent, which has made school seem that much more overbearing.”

Survey participants had the opportunity to elaborate on any answers from the standardized questionnaire. Fourteen out of 46 M1 participants reported feedback for free response Q11: “Please elaborate on any of the answers above.” Outstanding themes reflecting course change include (1) increased flexibility and efficiency, (2) mental health, (3) poor student/faculty relationships, (4) barriers and difficulties of online learning, and (5) lack of consistency.

Representative testimonials from students are illustrated in Table 2.

**Table 2:** Representative testimonials from free response. “Please elaborate on any of the answers above.”

**Theme**	**Quotes**
Increased flexibility and efficiency	I spent almost all of my study time at home, with an occasional day at campus. I found over time that my performance was improved through the use of high yield resources, as opposed to lectures. This may be because I was not able to ask questions and dive into specific topics that I needed more info on when it came to many of the pre-recorded lectures
	I was thankful to be able to be remote. It allowed me to help out my family (who don’t live in Vegas) with grocery shopping and other chores in order to reduce their risk of getting COVID. I didn’t have any trouble studying by myself. The best resources for M1 (BnB and question banks) are all available online anyways
	In terms of the normal academic courses, I think COVID made it easier to adjust to the workload of medical school, as I tend to study better in solitude. I don’t feel that it has negatively impacted the academic portion of medical school
	Even before covid I had planned to use the recorded lectures for study and to study solo. The ability to pause and rewind makes studying so much simpler because I can stop and think about it
	Personally, staying home saved me a lot of time and money
	COVID has allowed me to focus more on my studies, my hobbies, and my family. I have been very happy. School is stressful, but making my own schedule, and not wasting time in traffic has been immensely helpful to me
Mental health	[My] Mental health [was] greatly impacted due to lack of socializing
	Getting to know people without being there with them or having experiences with them is very hard whether that is faculty or my other colleagues
	Studying alone in your room day in and day out wears down on you over time
	I was (and still am) isolated from not only my friends, because I am in med school and I have to study, but also my classmates. I am totally alone
	COVID has taken away so many components from our educational experience and enrichment. It also left me struggling with mental health challenges on top of other difficult things in life. It’s left me with some long term side effects as well
	Very negatively. I’m someone who learns best from discussing in a small group and I live alone, so being at home was very isolating
	It has made other things I like doing outside of school either limited or non-existent, which has made school seem that much more overbearing
Poor student/faculty relationships	When it comes to knowing faculty, I’ve watched the classes above us interact with faculty and I do not feel as though I even have half of a relationship with anyone
	I haven’t been able to get to know faculty and classmates as well
	COVID has also severely limited my opportunities to connect and network with other students and faculty. I feel like I don’t have typical study friends like I had in undergrad
	It has made the faculty not trust students because they think we are just faces on the screen and not actual people
	My first year of medical school has been an all-around awful experience, 100% due to the virtual reality of COVID. I barely know most of my classmates
Barriers and difficulties of online learning	Discussion-based learning is very difficult for me to do on-camera and away from my colleagues. I started the year doing PBL in-person, and it was going really well; since we switched to online PBL, I have struggled with it
	I don’t know what abnormal lungs sound like or murmurs. We’ve been told to listen to our families but that doesn’t work for everyone. Not having in person FCP or PCC is only going to hurt us once LICs come around. We won’t be doing rotations online
	As for lectures, there were too many technological difficulties. It’s [also] extremely hard to learn how to do physical, tactile things online. I think being online for as long as we are has made me forget how great actual medicine is
	I do feel like I lack practical skills from no hands on experience when it comes to clinical practice. I found myself forgetting I was in medical school sometimes because of all the online aspects
	We missed out on quite a bit with the first 6 weeks being cancelled/moved (EMT) and now basically meaningless at this point if we end up doing it at the end of phase 1. Also having not touched/interacted with a real patient in person is hard for our education
	I do not feel I’m learning as effectively, and I think that not being on campus and physically present for the curriculum has led to some habits that in the long run aren’t very sustainable in terms of solidifying and mastering the material
Lack of consistency	In the fall, we were told we would be remote for the remainder of the year, then we got an email saying we had to start taking exams in person. I had to scramble to find housing and transportation to Vegas. It added a lot of unnecessary stress with no explanation as to why in-person testing was being implemented

## Discussion

The findings of this study showed significant differences between exam performance, student/faculty relationships, and study environments when comparing COVID and pre-COVID cohorts.

### Exam Performance

The most notable finding of this study was the difference in exam performance between the COVID cohort and pre-COVID cohorts. This is inconsistent with previous work where exam scores did not show significant differences between pre-COVID and COVID cohorts [9]. Furthermore, the students surveyed in this study had an overall positive view on the online format of their courses [9]. These findings suggest that student experience is greatly dependent on how medical schools transition to online learning and the format of their online courses. Both our study and the study performed by Grand et al. [9] evaluated the exam performance and overall medical student experience during COVID from a single institution. Future studies should evaluate the differences in curriculum between medical schools who had positive student experiences with schools who had negative student experiences to determine how to effectively teach courses in an online format.

### Location/Type of Study

Students in the COVID cohort spent significantly more time studying at home compared to students in the pre-COVID cohorts. This finding aligns with the COVID-19 pandemic causing the medical education curriculum to transition from live, in-person teaching to completely online in this cohort. Furthermore, students in the COVID cohort spent significantly more time studying alone vs. cohorts pre-COVID. The increased time spent at home seemed to contribute to the increased flexibility with remote learning, which students viewed to be a positive effect of the COVID-19 pandemic. This result is consistent with previous studies, showing medical students enjoy having increased flexibility during the COVID-19 pandemic [4]. Many of the students surveyed in our study enjoyed the ability to watch pre-recorded lectures on their own time and at their own pace. Furthermore, students reported they were able to spend more time with family and friends, save money, spend less time commuting, and more time enjoying other interests. Unfortunately, this study was not able to capture whether those who preferred virtual study environments performed better or worse than their counterparts due to design limitations as exam data and survey responses were collected separately and anonymously. However, these results do suggest that giving students the option to either come to campus for live lecture or stay home to watch pre-recorded lectures might be beneficial for individual student well-being. Future research could attempt to determine if this preference has any correlation with exam performance.

### Relationships Among Students and Faculty

Compared to non-COVID cohorts, COVID students reported weaker relationships with both their classmates and faculty. With their education moved online, students were restricted to socializing with classmates and faculty in virtual settings. The first semester of medical school is a difficult transition, in which many students rely on each other for support and seek assistance from faculty [2]. Therefore, the lack of in-person social interaction with classmates and faculty added to an overall negative experience. Schools who are thinking of switching to a virtual format should ensure enough opportunities are created for students and faculty members to get to know each other.

### Mental Health

Although mental health ratings in this study were not statistically significant, students did report an overall decline in mental health due to spending more time in isolation and much less time interacting with their peers, friends, and families as often as they did pre-pandemic. This feedback is consistent with previous literature that shows students during COVID-19 often feel detached from friends and family [10, 11]. This overall decline in mental health could be in part due to the shutdown of stress-relieving facilities such as gyms, clubs, movie theaters, etc. The Kirk Kerkorian School of Medicine at UNLV attempted to address this by hosting weekly outdoor lunches, increasing availability of virtual counseling sessions, and offering online “coffee dates” with faculty. Although these services were increased during COVID-19, they have always been available and utilized by previous classes. This might explain why there was no significant difference in overall mental health ratings between the COVID and pre-COVID cohorts. Future studies should evaluate which aspects of mental health were specifically affected by COVID-19 and should explore strategies on how to minimize the feeling of isolation for students who are taking classes online. Going forward, schools that switch to online learning should ensure that students have ways to continue interacting with faculty members and other students. These schools should also make sure they are regularly following up with their students and offer a variety of mental health services.

### Barriers to Online Learning

Several barriers to online learning contributed to students’ overall negative experience. These barriers were similar to ones assessed in previous studies and included unstable Internet connection, server outages, and content not being uploaded on time [6, 7]. Those with less reliable Internet access reported difficulty loading virtual conference calls for PBL, attending online live lectures, and streaming pre-recorded videos. These technical difficulties made it challenging for students to feel engaged and to learn without interruption. In the future, schools who are considering switching to online learning should make sure all students have access to reliable Internet and upload education material ahead of time. Furthermore, many students reported concerns with not being able to learn and practice clinical skills in-person. Although the clinical skills course was taught online, students felt they were not adequately prepared to conduct patient physical exams, including listening to lung and heart sounds. This lack of hands-on training potentially poses a risk of medical students being unprepared for their clerkships once they complete their preclinical years.

### Study Limitations

Limitations of this study include that it is a single-institution study. Similar studies will need to be conducted at other medical schools to determine the generalizability of the results. Furthermore, the authors did not gather pre-matriculation data, such as GPAs and MCAT scores, which would have provided relative academic aptitude between cohorts. According to the AAMC FACTS Application and Matriculation Data, nationwide averages in accepted MCAT and GPAs do not vary significantly from 2017 to 2021. We believe this consistency is applicable to the Kirk Kerkorian School of Medicine at UNLV, though this does remain a limitation. Finally, we note some limitations in the form of recall bias, due to survey items requiring participants to remember past experiences, as well as with selection bias since responses were voluntarily collected.

## Conclusion

Our study shows that during the COVID-19 pandemic, medical students at Kirk Kerkorian School of Medicine at UNLV have demonstrated decreased exam performance, study more at home, and have poorer relationships with their peers and faculty. Looking at all the variables, it is likely relationships exist between them. For example, study location and relationships may contribute to academic performance. Future studies are needed to determine if the next cohort, which has returned to pre-COVID in-person learning without any updates to their curriculum, will demonstrate academic performance and interpersonal behavior more similar to the COVID cohort or the pre-COVID cohort.
